# Eicosapentaenoic Acid Enhances the Effects of Mesenchymal Stromal Cell Therapy in Experimental Allergic Asthma

**DOI:** 10.3389/fimmu.2018.01147

**Published:** 2018-05-24

**Authors:** Soraia Carvalho Abreu, Miquéias Lopes-Pacheco, Adriana Lopes da Silva, Debora Gonçalves Xisto, Tainá Batista de Oliveira, Jamil Zola Kitoko, Lígia Lins de Castro, Natália Recardo Amorim, Vanessa Martins, Luisa H. A. Silva, Cassiano Felippe Gonçalves-de-Albuquerque, Hugo Caire de Castro Faria-Neto, Priscilla Christina Olsen, Daniel Jay Weiss, Marcelo Marcos Morales, Bruno Lourenço Diaz, Patricia Rieken Macêdo Rocco

**Affiliations:** ^1^Laboratory of Pulmonary Investigation, Carlos Chagas Filho Institute of Biophysics, Federal University of Rio de Janeiro, Rio de Janeiro, Brazil; ^2^Department of Medicine, College of Medicine, University of Vermont, Burlington, VT, United States; ^3^Laboratory of Cellular and Molecular Physiology, Carlos Chagas Filho Institute of Biophysics, Federal University of Rio de Janeiro, Rio de Janeiro, Brazil; ^4^Laboratory of Clinical Bacteriology and Immunology, School of Pharmacy, Federal University of Rio de Janeiro, Rio de Janeiro, Brazil; ^5^Laboratory of Inflammation, Carlos Chagas Filho Institute of Biophysics, Federal University of Rio de Janeiro, Rio de Janeiro, Brazil; ^6^Biomedical Institute, Federal University of the State of Rio de Janeiro, Rio de Janeiro, Brazil; ^7^Laboratory of Immunopharmacology, Oswaldo Cruz Institute, FIOCRUZ, Rio de Janeiro, Brazil; ^8^National Institute of Science and Technology for Regenerative Medicine, Rio de Janeiro, Brazil

**Keywords:** inflammation, remodeling, lung mechanics, histology, resolvin

## Abstract

Asthma is characterized by chronic lung inflammation and airway hyperresponsiveness. Despite recent advances in the understanding of its pathophysiology, asthma remains a major public health problem and, at present, there are no effective interventions capable of reversing airway remodeling. Mesenchymal stromal cell (MSC)-based therapy mitigates lung inflammation in experimental allergic asthma; however, its ability to reduce airway remodeling is limited. We aimed to investigate whether pre-treatment with eicosapentaenoic acid (EPA) potentiates the therapeutic properties of MSCs in experimental allergic asthma. Seventy-two C57BL/6 mice were used. House dust mite (HDM) extract was intranasally administered to induce severe allergic asthma in mice. Unstimulated or EPA-stimulated MSCs were administered intratracheally 24 h after final HDM challenge. Lung mechanics, histology, protein levels of biomarkers, and cellularity in bronchoalveolar lavage fluid (BALF), thymus, lymph nodes, and bone marrow were analyzed. Furthermore, the effects of EPA on lipid body formation and secretion of resolvin-D_1_ (RvD_1_), prostaglandin E_2_ (PGE_2_), interleukin (IL)-10, and transforming growth factor (TGF)-β1 by MSCs were evaluated *in vitro*. EPA-stimulated MSCs, compared to unstimulated MSCs, yielded greater therapeutic effects by further reducing bronchoconstriction, alveolar collapse, total cell counts (in BALF, bone marrow, and lymph nodes), and collagen fiber content in airways, while increasing IL-10 levels in BALF and M2 macrophage counts in lungs. In conclusion, EPA potentiated MSC-based therapy in experimental allergic asthma, leading to increased secretion of pro-resolution and anti-inflammatory mediators (RvD_1_, PGE_2_, IL-10, and TGF-β), modulation of macrophages toward an anti-inflammatory phenotype, and reduction in the remodeling process. Taken together, these modifications may explain the greater improvement in lung mechanics obtained. This may be a promising novel strategy to potentiate MSCs effects.

## Introduction

Allergic asthma is a chronic inflammatory disease characterized by airflow obstruction and airway hyperresponsiveness driven by immune responses to allergens (1). The imbalance between tissue injury and repair caused by chronic inflammation leads to the hallmark features of the chronic lung remodeling process in asthma (2). At present, no therapeutic approaches can reverse airway remodeling. Inhaled or systemic corticosteroids can decrease chronic inflammation, but do not halt or reverse the remodeling process in the lungs (3, 4), and long-term use of high-dose corticosteroids can cause systemic side effects (5). Therefore, there is an unmet need for novel therapeutic strategies that can repair damaged tissue, while simultaneously mitigating the inflammation and remodeling process.

Several studies have demonstrated that mesenchymal stromal cells (MSCs) have strong immunomodulatory properties and are able to secrete soluble paracrine factors (6–8). Systemic or intratracheal administration of MSCs derived from bone marrow, adipose, and other tissues has been shown to significantly reduce inflammation and improve airway hyperresponsiveness in different models of allergic asthma (6, 9–11). However, their ability to reverse airways remodeling is only marginal (7, 12, 13). Given this limitation, recent studies have tried to potentiate the therapeutic effects of MSCs by using physical, biological, and/or chemical pre-stimulation to enhance cell survival and regenerative properties (14–17).

Omega-3 fatty acids are polyunsaturated essential fatty acids, mainly found in fish oil, that have immunomodulatory properties (18, 19). In particular, eicosapentaenoic acid (EPA) has been found to inhibit inflammatory responses in human asthmatic alveolar macrophages more efficiently than docosahexaenoic acid (20). Some reports have also shown that EPA reduces mucus hypersecretion and levels of several inflammatory mediators as well as can enhance the regulatory T-cell response (21). Additionally, EPA serves as a substrate during cellular stress to produce anti-inflammatory mediators, such as eicosanoids and resolvins (18), and suppress tissue remodeling by reducing collagen deposition in the airways (22). However, the effects of EPA on MSCs remain unknown, as well as whether pre-incubation of MSCs with EPA could enhance the therapeutic effects of MSCs in asthma.

The present study aimed to investigate whether exposure of MSCs to EPA could potentiate their effects in experimental allergic asthma by enabling them to further reduce inflammation and airway remodeling. For this purpose, unstimulated and EPA-stimulated MSCs were administered in a murine model of house dust mite (HDM)-induced allergic asthma to comparatively evaluate their therapeutic effects on lung mechanics, histology, protein levels of pro-inflammatory biomarkers and cellularity in the bronchoalveolar lavage fluid (BALF), thymus, mediastinal lymph nodes (mLNs), and bone marrow. Furthermore, the effects of EPA on lipid body formation, and interleukin (IL)-10, transforming growth factor (TGF)-β1, resolvin-D_1_ (RvD_1_), and prostaglandin E_2_ (PGE_2_) secretion by MSCs were evaluated *in vitro*.

## Materials and Methods

### Experimental Protocol

Seventy-two C57BL/6 mice (64 females and 8 males, weight 20–25 g, age 8–10 weeks) were used. MSCs were harvested from male mice and characterized. Thirty-two females were used to evaluate lung mechanics and histology, while the remaining 32 were used to analyze biomarker secretion, total and differential cell counts in BALF, and cell counts in bone marrow, lymph nodes, and thymus (*n* = 8/group).

All animals were randomly allocated across two groups (Figure S1 in Supplementary Material). In the HDM group, mice were challenged with intranasal instillation of 25 µg protein (diluted in 25 µL of phosphate-buffered saline [PBS]) presented in HDM extract, on 3 days/week for 3 weeks (13, 23). The control (CTRL) group received intranasal instillation of sterile PBS under the same protocol. Twenty-four hours after the last challenge, the HDM group was subsequently randomized into three subgroups to receive sterile saline (50 µL, SAL) or MSCs (10^5^ cells per mouse) unstimulated or stimulated with EPA *via* the intratracheal route. Three days after therapy, mice were euthanized, and all data analyzed. Investigators were blinded to experimental groups for all *in vivo* and *in vitro* measurements.

### MSCs Stimulation and Characterization

Male C57BL/6 mice (weight 20–25 g, age 8–10 weeks) were anesthetized with intravenous ketamine (25 mg/kg) and xylazine (2 mg/kg) and used as cell donors. Bone marrow cells were obtained from femurs and tibias as described (7, 24). After isolation, bone marrow-derived cells were cultured (37°C, 5% CO_2_ in humidified atmosphere) with Dulbecco’s Modified Eagle Medium (DMEM; Invitrogen, CA, USA) containing 15 mM HEPES (Sigma, MO, USA), 15% inactivated fetal bovine serum (FBS) (Invitrogen, CA, USA), 100 U/mL penicillin, and 100 mg/mL streptomycin antibiotic solution (Gibco, NM, USA). Upon reaching 80% confluence, adherent cells were passaged with 0.05% trypsin-EDTA solution (Gibco, NM, USA) and then maintained in DMEM with 10% FBS and penicillin/streptomycin. Third-passage MSCs were stimulated for 6 h with EPA (10 µM, CAS 10417-94-4, Cayman Chemical, Ann Arbor, MI, USA), and then washed in 1× PBS and trypsinized. Viable cells were concentrated at 1 × 10^5^ in 50 µL of sterile saline solution for therapeutic injection. MSCs were characterized on the basis of the following criteria: (1) MSCs must be plastic-adherent when maintained in standard culture conditions using tissue culture flasks; and (2) 95% of the MSC population must express specific surface antigens. MSCs were phenotyped by flow cytometry using commercially available antibodies against CD24 (heat stable antigen), CD31 (endothelial cell marker), CD44 (hyaluronic acid receptor), CD45 (hematopoietic marker), CD49e (integrin alpha-5), MHC class II, and stem cell antigen-1 (Sca-1) (all from BD Biosciences, USA). All data were acquired in a FACSCalibur flow cytometer (Becton Dickinson Biosciences Immunocytometry Systems, San Jose, CA, USA) and analyzed using FlowJo X 10.0.7 software (Tree Star Inc., Ashland, OR, USA).

### Lung Mechanics

Three days after saline or MSCs administration, the animals were sedated (diazepam 1 mg/kg intraperitoneally), anesthetized (thiopental sodium 20 mg/kg intraperitoneally), tracheotomized, paralyzed (vecuronium bromide, 0.005 mg/kg intravenously), and ventilated using a constant-flow ventilator (Samay VR15; Universidad de la Republica, Montevideo, Uruguay) with the following settings: frequency 100 breaths/min, tidal volume (V_T_) 0.2 mL, and fraction of inspired oxygen 0.21. The anterior chest wall was surgically removed and a positive end-expiratory pressure (PEEP) of 2 cmH_2_O applied. Airflow and tracheal pressure (Ptr) were measured (25). Lung mechanics were analyzed by the end-inflation occlusion method (26). In an open chest preparation, Ptr reflects transpulmonary pressure (P_L_). Briefly, after end-inspiratory occlusion, there is an initial, precipitous drop in P_L_ (ΔP1,L) from the pre-occlusion value down to an inflection point (Pi), followed by a slow pressure decay (ΔP2,L), until a plateau is reached. This plateau corresponds to the elastic recoil pressure of the lung (Pel). ΔP1,L selectively reflects the pressure used to overcome the airway resistance. ΔP2,L reproduces the pressure spent by stress relaxation, or viscoelastic properties of the lung, together with a small contribution of *pendelluft*. Static lung elastance (Est,L) was determined by dividing Pel by V_T_. Lung mechanics were measured 10 times in each animal (27). All data were analyzed using ANADAT software (RHT-InfoData, Inc., Montreal, QC, Canada).

### Lung Histology

Immediately after determination of lung mechanics, laparotomy was performed and heparin (1,000 IU) was injected into the vena cava. The trachea was clamped at end-expiration (PEEP = 2 cmH_2_O), and the abdominal aorta and vena cava were transected to cause death by exsanguination. Lungs were then removed and flash-frozen by immersion in liquid nitrogen. The left lung was fixed with Carnoy’s solution and paraffin-embedded (28). Sections (4-µm thick) were cut and stained with hematoxylin-eosin for morphometric analysis of lung structure (27, 29). Lung morphometry analysis was performed using an integrating eyepiece with a coherent system consisting of a grid with 100 points and 50 lines of known length coupled to a conventional light microscope (Olympus BX51, Olympus Latin America-Inc., Brazil). The volume fraction of collapsed and normal pulmonary areas and magnitude of bronchoconstriction were determined by the point-counting technique (7, 27) across 10 random, non-coincident fields of view per mouse. Specific staining methods to quantify elastic and collagen fibers (Weigert’s resorcin-fuchsin method with oxidation and Masson’s trichome method, respectively) were also used. These fibers were quantified in airways and alveolar septa using ImagePro Plus 6.0 software (7). Data are expressed as percentage of elastic and collagen fibers per total tissue area. Finally, the presence of mucus-filled cells in each airway was revealed by periodic acid–Schiff-staining and scored on a scale of 0–4. The average of values obtained from 6 to 10 airways was taken as the overall mucus score per mouse, as previously described (13).

### Immunohistochemistry for α-Smooth Muscle Actin (SMA)

Right lungs were fixed and embedded in paraffin for immunohistochemistry using a monoclonal antibody against α-smooth muscle actin (α-SMA; Dako, Carpinteria, CA, USA) at a 1:500 dilution. Analysis was performed by application of the point-counting technique (29). Using a 121-point grid, the volume proportion of α-SMA was calculated as the ratio of points falling on actin-stained vs. non-stained tissue.

### Immunohistochemistry for Analysis of Macrophage Phenotype

Sections from the right lungs were deparaffinized and hydrated, and the slides incubated with 10 mM sodium citrate. Endogenous peroxidase activity was blocked with 3% hydrogen peroxide. Slides were washed in TBS with 0.05% Tween-20 (Sigma, St. Louis, MO, USA), blocked with serum-free protein block (Dako, Carpinteria, CA, USA), and immunostained with Vectastain ABC (Vector Laboratories, Inc., Burlingame, CA, USA). This was followed by incubation with antibodies for CD68 (1:100 dilution, total macrophage marker), iNOS (1:350 dilution, M1 marker), and CD163 (1:100 dilution, M2 marker) in TBS/Tween buffer, overnight at 4°C. Staining was developed with 3,3′-diaminobenzidine tetrahydrochloride (Vector Laboratories, Inc.) and counterstained with hematoxylin. An isotype immunoglobulin G was used as negative control (24, 30). Data are presented as percentage of macrophages stained positive for iNOS and CD163 per total number of macrophages (CD68^+^) in lung tissue.

### Total and Differential Cell Count

#### Bronchoalveolar Lavage Fluid

Bronchoalveolar lavage fluid was obtained by gentle aspiration of 400 mL of PBS 1× (final volume 1.2 mL) injected into the airways *via* a tracheal cannula. BALF was centrifuged at 250 *g* for 10 min at 4°C. Cell pellets were resuspended in PBS 1×.

#### Bone Marrow

Bone marrow was obtained by gentle lavage of the right femur of each animal with 1 mL of PBS 1×.

#### Lymph Nodes

Mediastinal lymph nodes were carefully extracted and placed in 1 mL of PBS 1×. Cell suspensions were obtained after mechanical homogenization.

#### Thymus

The thymus of each animal was carefully extracted and placed in 1 mL of PBS 1×. Again, cell suspensions were obtained after mechanical homogenization.

Total leukocytes from BALF, bone marrow, lymph nodes, and thymus were obtained as previous described (23) and then counted in a Neubauer chamber after dilution with Turk’s solution (2% acetic acid). Thereafter, BALF and bone marrow cells were pelleted onto glass slides by cytocentrifugation and stained by the May–Grünwald–Giemsa method for differential cell counts as described elsewhere (7, 23).

### Lipid Body Staining and Counting

Mesenchymal stromal cells were cultured in 12-well plate (10^5^ cells/well) for 24 h. Subsequently, cells were stimulated or not with EPA for 6 h. After stimulation, MSCs were fixed in 3.7% formaldehyde Ca^2+^/Mg^2+^-free HBSS medium (pH 7.4) and stained with 1.5% OsO_4_, as previously described (31). Lipid bodies were enumerated by light microscopy using an oil-immersion objective lens in 50 consecutively scanned MSCs.

### Analysis of Multiple Soluble Factors *In Vivo* and After *In Vitro* Stimulation

Levels of vascular endothelial growth factor (VEGF), IL-4, IL-13 (PeproTech, Rocky Hill, NJ, USA), and IL-10 (Biolegend, San Diego, CA, USA) were quantified by ELISA in BALF. Additionally, levels of IL-10, TGF-β, RvD1, and PGE_2_ were quantified in the supernatants of unstimulated or EPA-stimulated MSCs. For this purpose, *in vitro* experiments were performed: 10^5^ MSCs were added to a 12-well plate and cultured for 24 h under normal conditions (DMEM–High Glucose medium supplemented with 10% FBS, 1,000 U/mL penicillin/streptomycin, and 2 mM l-glutamine; Invitrogen Life Technologies, Grand Island, NY, USA). The next day, MSCs received conditioned medium (FBS-free) and were stimulated or not with EPA for 6 h. After stimulation, the supernatants were collected and levels of IL-10, TGF-β (Biolegend, San Diego, CA, USA), RvD1, and PGE_2_ (Cayman Chemical, Ann Arbor, MI, USA) were analyzed by ELISA or EIA, respectively, as per manufacturer instructions. The 15-lipoxygenase inhibitor nordihydroguaiaretic acid (15-LO inhibitor 1, Cayman Chemical, Ann Harbor, MI, USA, 20 ng/mL) was added to culture medium to confirm the EPA-induced lipid mediator production. Results are expressed as pg/mL.

### Y-Chromosome DNA Detection

Three days after MSCs administration, Y-chromosome DNA in lung tissue was quantified by real-time polymerase chain reaction, as described elsewhere (30, 32).

### Statistical Analysis

Sample size was based on pilot studies and on our experience with models of allergic asthma (7, 13). Data were tested for normality using the Kolmogorov–Smirnov test with Lilliefors’ correction, while the Levene median test was used to evaluate homogeneity of variances. If both conditions were satisfied, differences between groups at *in vivo* analysis were assessed using one-way ANOVA followed by Tukey’s test. For nonparametric results, the Kruskal–Wallis test followed by Dunn’s test was used. For results of *in vitro* analysis, the Student’s *t*-test and Mann–Whitney *U* test were used as appropriate. Parametric data were expressed as mean ± SD, while nonparametric data were expressed as median (interquartile range). All tests were carried out in GraphPad Prism version 6.07 (GraphPad Software, La Jolla, CA, USA). Significance was established at *p* < 0.05.

## Results

### EPA Affected Neither MSC Morphology Nor MSC-Specific Cell-Surface Markers

Unstimulated and EPA-stimulated MSCs similarly displayed the characteristic spindle-shaped morphology, adherence to plastic culture dishes, and expression of MSC-specific cell-surface markers by flow cytometry (similarly negative for CD31, CD45, and MHCII and positive for CD24, CD44, CD49e, and Sca-1), ruling out any possibility that these cells could be fibroblasts (Figure S2 in Supplementary Material). Considering that the differentiation potential of unstimulated MSCs had been previously characterized (7, 13, 24) and that EPA-stimulated MSCs exhibited the characteristic morphology, adherence, and surface-marker expression profile, we did not evaluate the differentiation potential of EPA-stimulated MSCs into osteocytes, chondrocytes, and adipocytes.

### EPA Stimulated Lipid Body Formation in MSCs and Modulated Biomarker Secretion by MSCs

**Figure 1:**
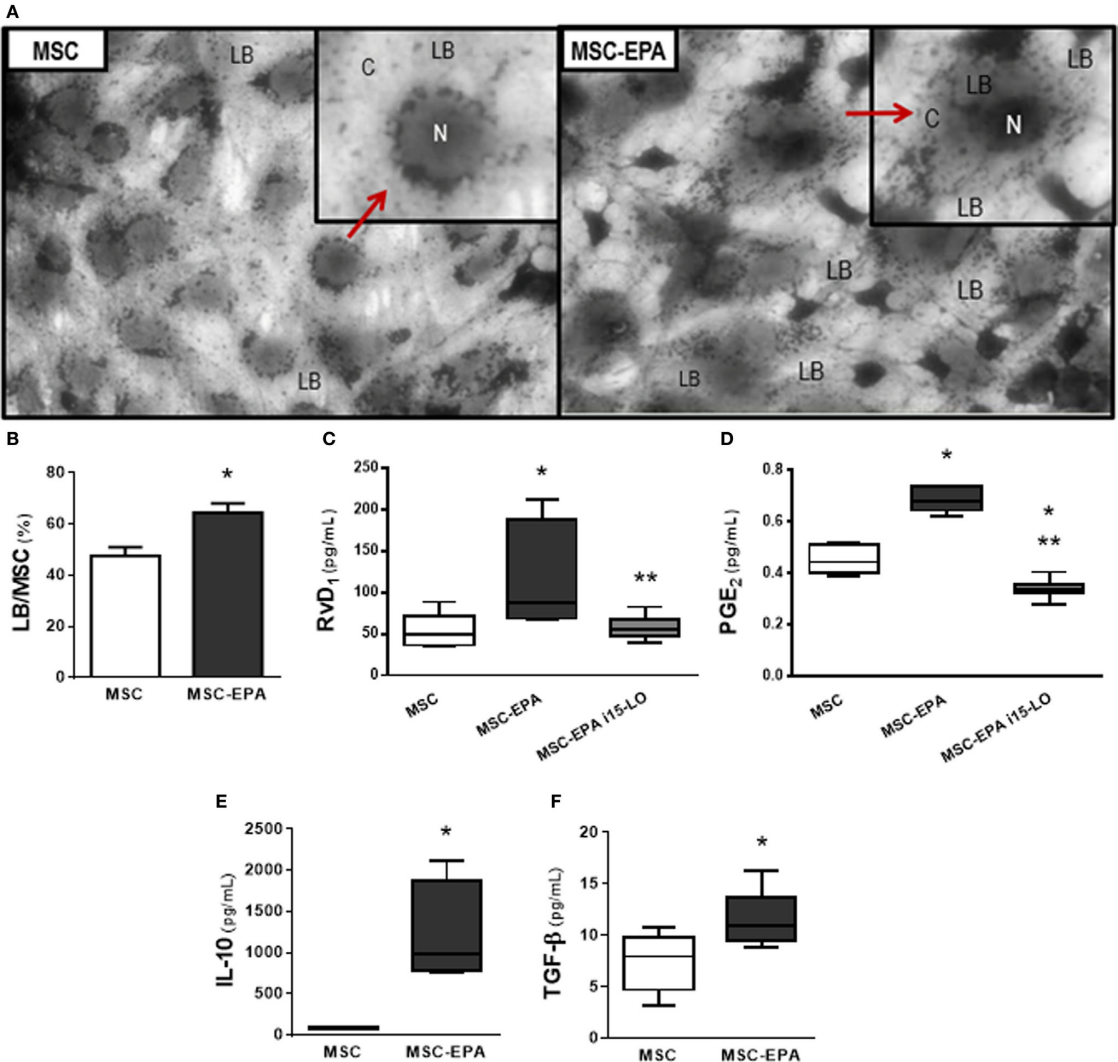
EPA-stimulated lipid body formation in MSCs and modulated secretion of biomarkers by MSCs. **(A)** LB (red arrow) in MSCs and EPA-MSCs stained with OsO_4_. **(B)** Quantification of LB per MSC. Levels of **(C)** RvD_1_ and **(D)** PGE_2_, assessed by EIA, and **(E)** IL-10 and **(F)** TGF-β, assessed by ELISA, in cells stimulated or not with EPA for 6 h. MSC, unstimulated MSCs; MSC-EPA, EPA-stimulated MSCs. Student’s *t-*test **(B)**, Kruskal–Wallis test followed by Dunn’s test **(C,D)**, and Mann–Whitney *U* **(E,F)** were used for statistical comparison. **(B)** Data presented as mean + SD of five independent experiments. **(C–F)** Boxes show the interquartile (25–75%) range, whiskers denote the range (minimum–maximum), and horizontal lines represent the median of five independent experiments. *Significantly different from MSC (*p* < 0.05). **Significantly different from MSC-EPA (*p* < 0.05). Abbreviations: EPA, eicosapentaenoic acid; MSCs, mesenchymal stromal cells; LB, lipid bodies; N, nucleus; C, cytoplasm; RvD_1_, resolvin D_1_; PGE_2_, prostaglandin E_2_; IL, interleukin; TGF-β, transforming growth factor-β; i15-LO, inhibitor of 15-lipoxygenase.

LBs are dynamic and functionally active cytosolic organelles in which polyunsaturated fatty acids and enzymes responsible for their metabolism congregate to produce lipid mediators such as eicosanoids (31, 33). Compared to unstimulated MSCs, EPA-stimulated MSCs presented higher LB count (1.35-fold increase), as well as increased levels of RvD_1_ (2.17-fold increase) and PGE_2_ (1.51-fold increase) (Figures 1A–D). Administration of 15-LO inhibition (i15-LO) abrogated the increased in RvD_1_ levels in EPA-stimulated MSCs, confirming the central role of 15-LO in RvD1 generation. Increased PGE_2_ levels were also inhibited by i15-LO, suggesting an autocrine role for its metabolites in the promotion of PGE_2_ generation. Increased production of RvD_1_ suggests a more pronounced anti-inflammatory profile. In fact, EPA-stimulated MSCs produced increased levels of IL-10 and TGF-β compared to unstimulated MSCs (14- and 1.56-fold increase, respectively) (Figures 1E,F).

### EPA-Stimulated MSCs Led to Greater Modulation of Biomarker Secretion and Reduction in BALF Cellularity Than Unstimulated MSCs

**Figure 2:**
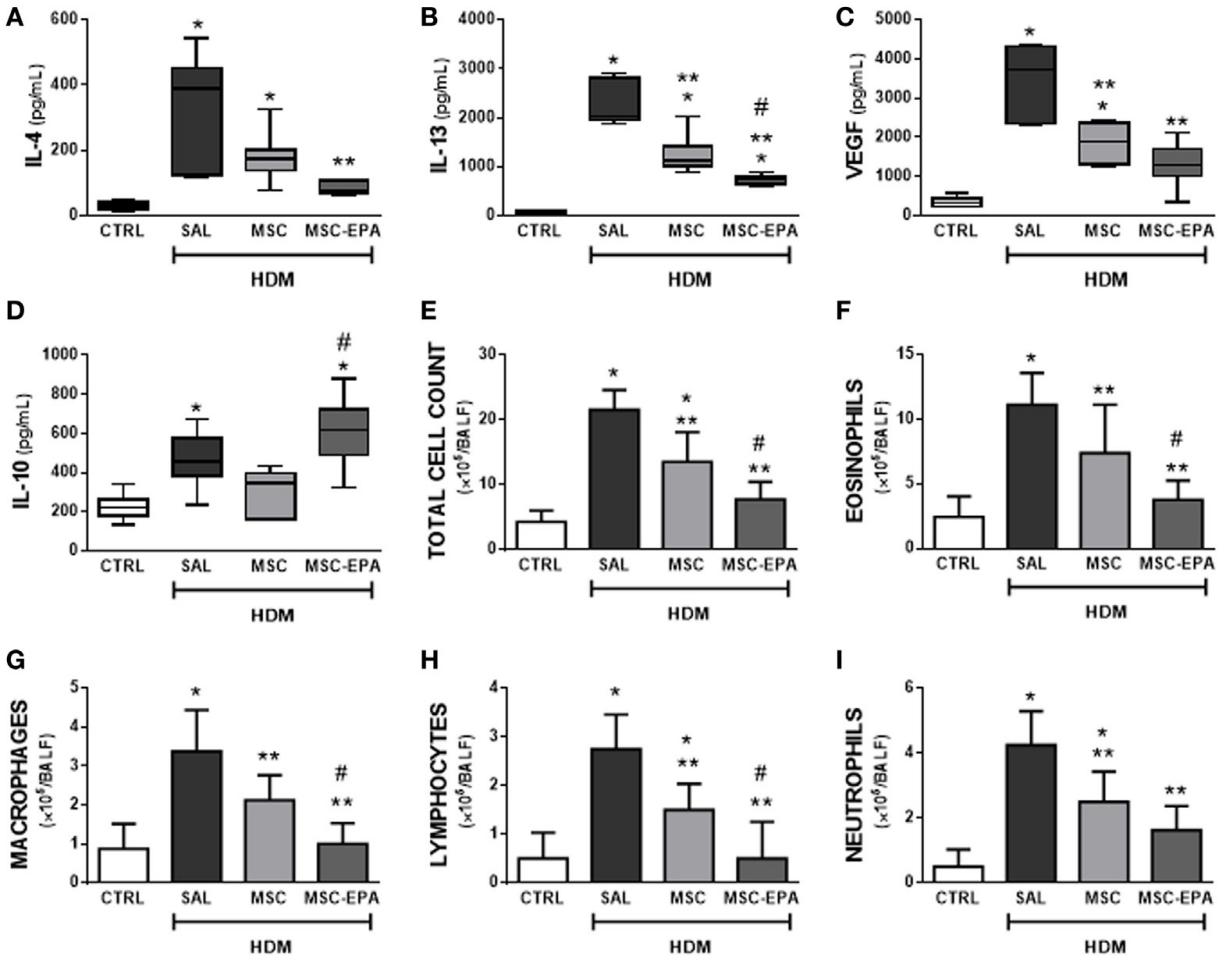
EPA-stimulated MSCs led to greater modulation of biomarker secretion and reduction in bronchoalveolar lavage fluid (BALF) cellularity than unstimulated MSCs. Protein levels of **(A)** IL-4, **(B)** IL-13, **(C)** VEGF, **(D)** IL-10, **(E)** total leukocytes, **(F)** eosinophils, **(G)** macrophages, **(H)** lymphocytes, and **(I)** neutrophil counts in BALF. CTRL, saline-challenged mice; HDM, HDM-challenged mice; SAL, HDM mice treated with saline; MSC, HDM mice treated with unstimulated MSCs; MSC-EPA, HDM mice treated with EPA-stimulated MSCs. Kruskal–Wallis test followed by Dunn’s test **(A–D)** and one-way ANOVA followed by Tukey’s test **(E–I)** were used for statistical comparison. **(A–D)** Boxes show the interquartile (25–75%) range, whiskers denote the range (minimum–maximum), and horizontal lines represent the median of eight animals/group. **(E–G)** Data presented as mean + SD of eight animals/group. *Significantly different from CTRL (*p* < 0.05). **Significantly different from HDM-SAL (*p* < 0.05). ^#^Significantly different from HDM-MSC (*p* < 0.05). Abbreviations: EPA, eicosapentaenoic acid; MSCs, mesenchymal stromal cells; HDM, house dust mite; IL, interleukin; VEGF, vascular endothelial growth factor.

The HDM-SAL group demonstrated higher levels of IL-4, IL-13, VEGF, and IL-10 in BALF than the CTRL group (Figures 2A–D). The HDM-MSC group demonstrated lower BALF levels of IL-13 and VEGF compared to the HDM-SAL group. Compared to HDM-MSC animals, HDM-MSC-EPA mice demonstrated an even greater reduction in protein levels of IL-13 and VEGF, as well as a reduction in IL-4, and an increase in IL-10 level in BALF. Total and differential cell counts in BALF were higher in HDM-SAL mice than in CTRL mice (Figures 2E–I). Both the HDM-MSC and HDM-MSC-EPA groups showed reductions in total and differential cellularity in BALF compared to HDM-SAL; however, reductions were more pronounced in HDM-MSC-EPA mice. Stimulation of MSCs with EPA produced an increase in their capacity to modulate inflammatory markers in HDM-challenged mice, strengthening the *in vitro* findings.

### EPA-Stimulated MSCs Induced Macrophage Polarization to the M2 Rather Than the M1 Profile

**Figure 3:**
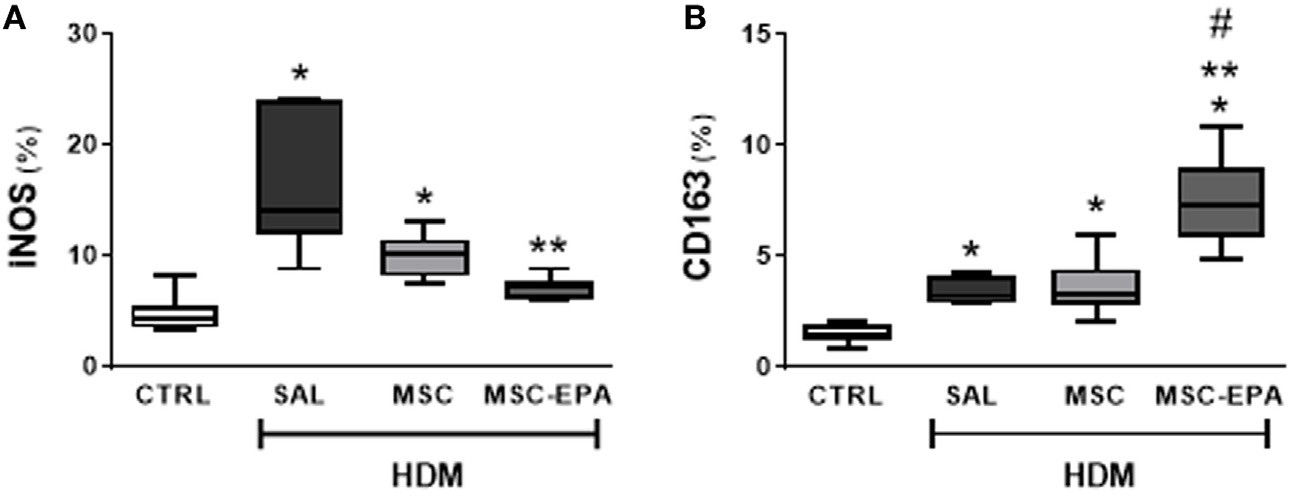
EPA-stimulated MSCs induced macrophage polarization toward an M2 rather than M1 profile. **(A)** M1-macrophage (iNOS^+^) and **(B)** M2-macrophage (CD163^+^) counts in lung tissue. CTRL, saline-challenge mice; HDM, HDM-challenged mice; SAL, HDM mice treated with saline; MSC, HDM mice treated with unstimulated MSCs; MSC-EPA, HDM mice treated with EPA-stimulated MSCs. The Kruskal–Wallis test followed by Dunn’s test was used for statistical comparison. Boxes show the interquartile (25–75%) range, whiskers denote the range (minimum–maximum), and horizontal lines represent the median of eight animals/group. *Significantly different from CTRL (*p* < 0.05). **Significantly different from HDM-SAL (*p* < 0.05). ^#^Significantly different from HDM-MSC (*p* < 0.05). Abbreviations: EPA, eicosapentaenoic acid; HDM, house dust mite; MSCs, mesenchymal stromal cells.

To evaluate macrophage polarization as a potential anti-inflammatory mechanism for EPA-stimulated MSCs, expressions of the M1 marker, iNOS, and the M2 marker, CD163, were analyzed. iNOS- and CD163-positive cell counts were increased in the lung sections of HDM-SAL animals as compared to CTRL animals (Figure 3; Figure S3 in Supplementary Material). HDM-SAL and HDM-MSC mice presented similar iNOS- and CD163-positive cell counts in lung tissue. In contrast, the CD163-positive cell count was increased, and the iNOS-positive cell count decreased in HDM-MSC-EPA group.

### EPA-Stimulated MSCs Led to Greater Reduction in Bone Marrow, Lymph Node, and Thymus Cellularity Than Unstimulated MSCs

**Figure 4:**
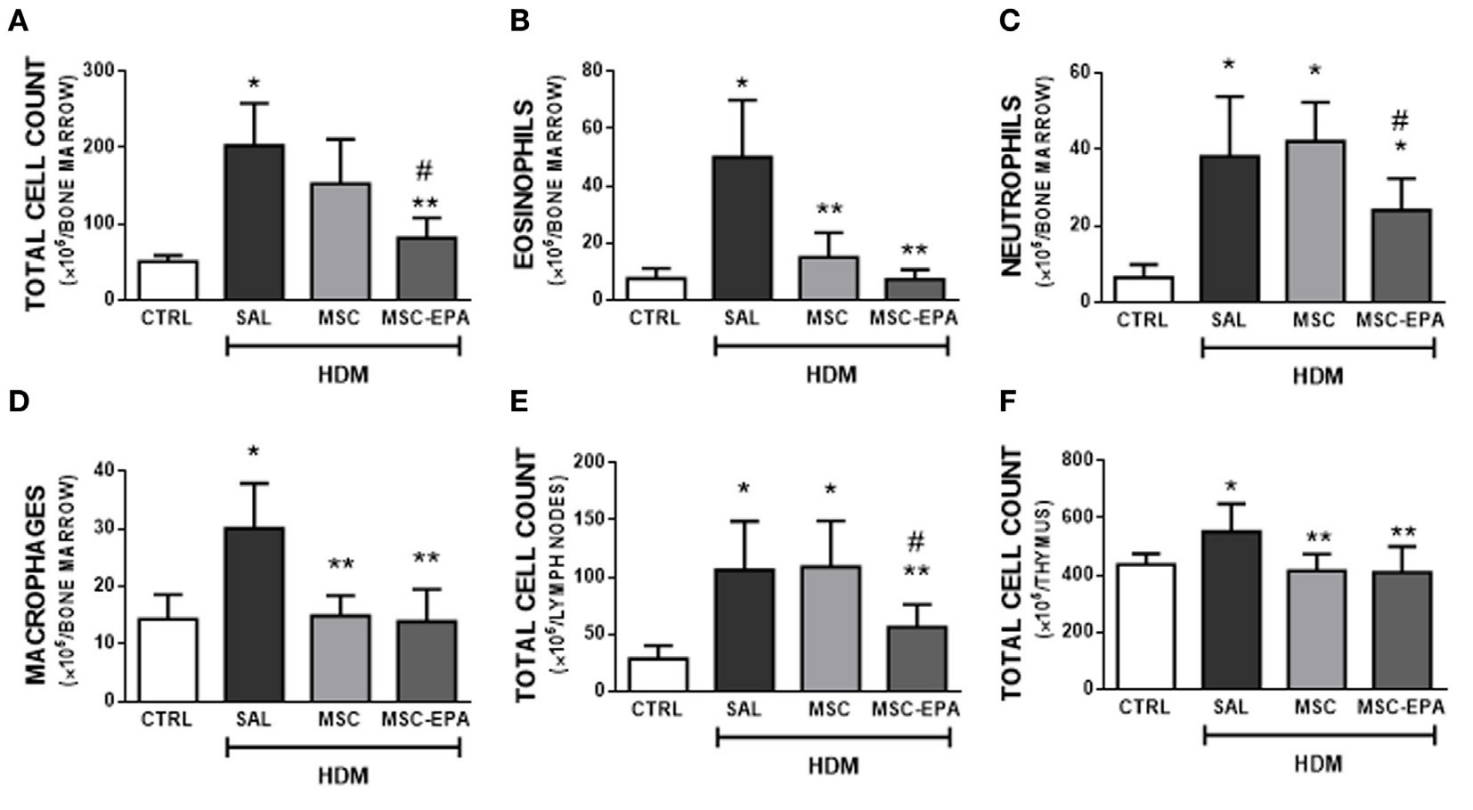
EPA-stimulated MSCs led to greater reductions in bone marrow, lymph nodes, and thymus cellularity than unstimulated MSCs. **(A)** Total leukocytes, **(B)** eosinophils, **(C)** neutrophils, **(D)** macrophages in bone marrow, **(E)** total leukocytes in mediastinal lymph nodes, and **(F)** total leukocytes in thymus. CTRL, saline-challenged mice; HDM, HDM-challenged mice; SAL, HDM mice treated with saline; MSC, HDM mice treated with unstimulated MSCs; MSC-EPA, HDM mice treated with EPA-stimulated MSCs. One-way ANOVA followed by Tukey’s test was used for statistical comparison. Data are presented as mean + SD. *n* = 8 animals/group. *Significantly different from CTRL (*p* < 0.05). **Significantly different from HDM-SAL (*p* < 0.05). ^#^Significantly different from HDM-MSC (*p* < 0.05). Abbreviations: EPA, eicosapentaenoic acid; MSCs, mesenchymal stromal cells; HDM, house dust mite.

HDM-SAL mice presented higher total cell count in bone marrow (Figure 4A), mLNs (Figure 4E), and thymus (Figure 4F) compared to CTRL animals. The HDM-MSC and HDM-MSC-EPA groups had similarly reduction in eosinophil and macrophage counts in bone marrow (Figures 4B,D) and total cell count in thymus (Figure 4F), comparable to those of CTRL mice. However, only the HDM-MSC-EPA group presented a decrease in total cell and neutrophil counts in bone marrow (Figures 4A,C) and total cell count in lymph nodes (Figure 4E).

### EPA-Stimulated MSCs Led to Greater Reductions in Lung Morphological Changes, Remodeling, and Mucus Hypersecretion Than Unstimulated MSCs

The HDM-SAL and HDM-MSC groups demonstrated a higher fractional area of alveolar collapse and bronchoconstriction index compared to CTRL (Table 1; Figure S4 in Supplementary Material). These morphological changes were only reduced in HDM-MSC-EPA mice, which exhibited levels similar to those of CTRL mice.

**Table 1:** Lung morphometry.

Groups		Normal (%)	Collapse (%)	Contraction index
CTRL		97.67 ± 1.32	2.33 ± 1.32	2.45 ± 0.24
HDM	SAL	92.65 ± 2.32*	7.15 ± 2.32*	3.69 ± 0.62*
	MSC	95.84 ± 1.14*	4.16 ± 1.14*	3.39 ± 0.25*
	MSC-EPA	97.21 ± 0.63**	2.79 ± 0.63**	2.97 ± 0.41**

*The volume fraction of collapsed and normal pulmonary areas and the magnitude of bronchoconstriction (contraction index). CTRL, saline-challenged mice; HDM, HDM-challenged mice; SAL, HDM mice treated with saline; MSC, HDM mice treated with unstimulated MSCs; MSC-EPA, HDM mice treated with EPA-stimulated MSCs. One-way ANOVA followed by Tukey’s test was used for statistical comparison. Data are presented as mean ± SD of eight animals/group*.

*Significantly different from CTRL (*p* < 0.05).

**Significantly different from HDM-SAL (*p* < 0.05).

**Figure 5:**
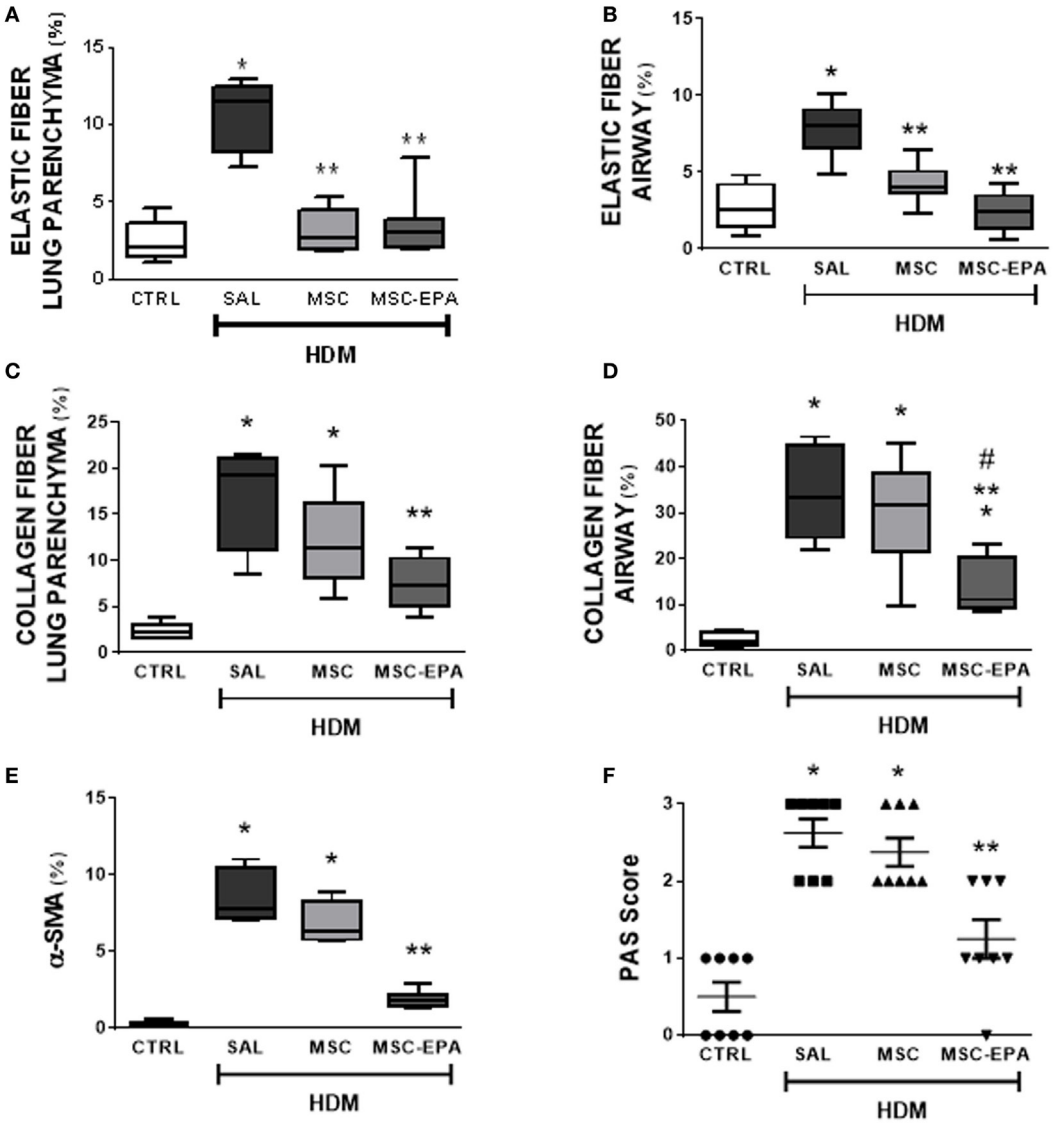
EPA-stimulated MSCs led to greater reductions in lung remodeling and mucus hypersecretion than unstimulated MSCs. Elastic fiber content in **(A)** lung parenchyma and **(B)** airway, collagen fiber content in **(C)** lung parenchyma and **(D)** airway, **(E)** α-SMA expression, and **(F)** mucus-filled cell count in lung tissue. CTRL, saline-challenged mice; HDM, HDM-challenged mice; SAL, HDM mice treated with saline; MSC, HDM mice treated with unstimulated MSCs; MSC-EPA, HDM mice treated with EPA-stimulated MSCs. The Kruskal–Wallis test followed by Dunn’s test was used for statistical comparison. Boxes show the interquartile (25–75%) range, whiskers denote the range (minimum–maximum), and horizontal lines represent the median of eight animals/group. *Significantly different from CTRL (*p* < 0.05). **Significantly different from HDM-SAL (*p* < 0.05). ^#^Significantly different from HDM-MSC (*p* < 0.05). Abbreviations: EPA, eicosapentaenoic acid; MSCs, mesenchymal stromal cells; HDM, house dust mite; SMA, smooth muscle actin.

Compared to CTRL group, the HDM-SAL group demonstrated increased elastic and collagen fiber content in alveolar septa and airways, as well as increased α-SMA expression in lung tissue (Figures 5A–E; Figure S5 in Supplementary Material). Elastic fiber content was similarly reduced in HDM-MSC and HDM-MSC-EPA mice compared to HDM-SAL mice (Figures 5A,B). In addition, the HDM-MSC-EPA group, but not the HDM-MSC group, exhibited lower collagen fiber content (Figures 5C,D) as well as α-SMA expression (Figure 5E) in lung tissue.

Very few mucin-containing cells were observed in the lung tissue of CTRL mice, whereas the number of cells was significantly increased in the HDM-SAL group (Figure 5F). HDM-MSC-EPA mice, but not HDM-MSC mice, demonstrated a decreased number of mucin-containing cells.

### EPA-Stimulated MSCs Led to Greater Improvement in Lung Mechanics Than Unstimulated MSCs

**Figure 6:**
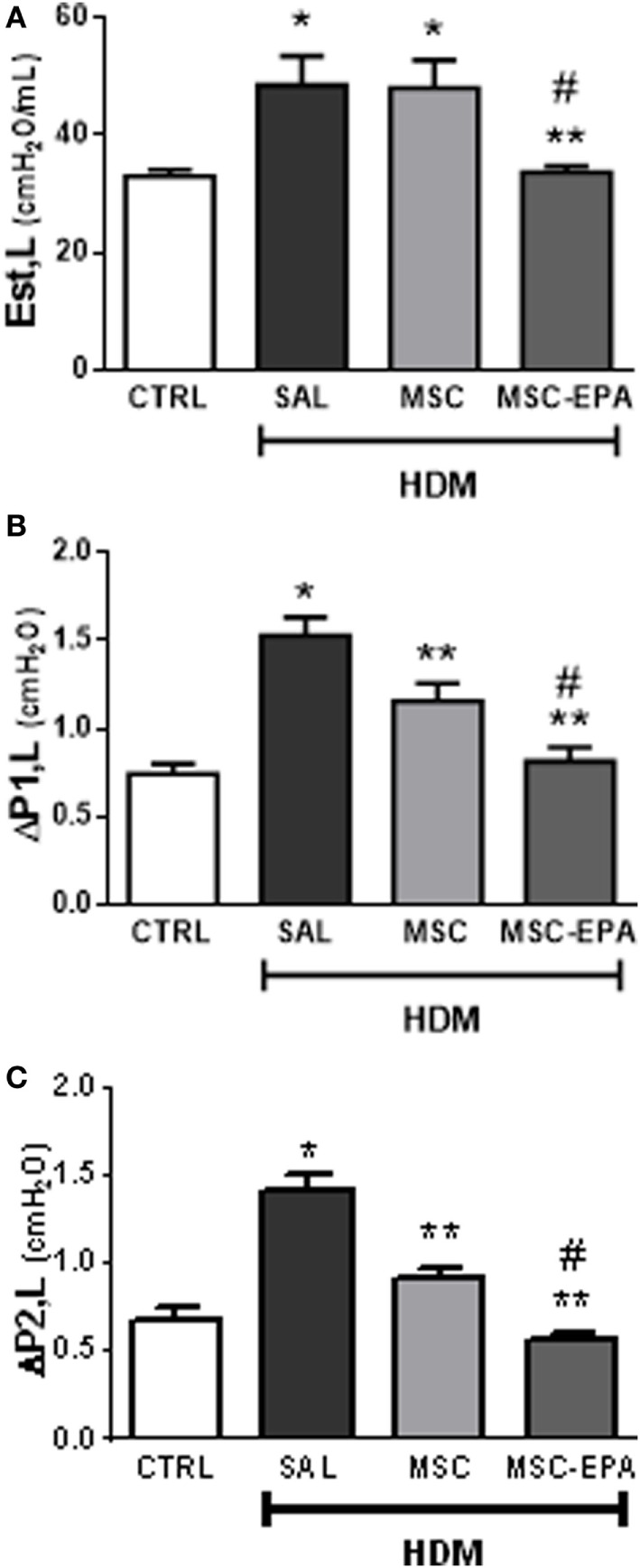
EPA-stimulated MSCs led to greater improvement in lung mechanics than unstimulated MSCs. **(A)** Static lung elastance (Est,L), **(B)** resistive (ΔP1,L) pressure, and **(C)** viscoelastic (ΔP2,L) pressure. CTRL, saline-challenged mice; HMD, HMD-challenged mice; SAL, HDM mice treated with saline; MSC, HDM mice treated with unstimulated MSCs; MSC-EPA, HDM mice treated with EPA-stimulated MSCs. One-way ANOVA followed by Tukey’s test was used for statistical comparison. Data are presented as mean + SD. *n* = 8 animals/group. *Significantly different from CTRL (*p* < 0.05). **Significantly different from HDM-SAL (*p* < 0.05). ^#^Significantly different from HDM-MSC (*p* < 0.05). Abbreviations: EPA, eicosapentaenoic acid; MSCs, mesenchymal stromal cells; HDM, house dust mite.

The HDM-SAL group demonstrated higher Est,L, ΔP1,L, and ΔP2,L (Figures 6A–C) compared to the CTRL group (1.47-, 2.06-, and 2.11-fold increase, respectively). The HDM-MSC and HDM-MSC-EPA groups exhibited decreased ΔP1,L and ΔP2,L; however, these reductions were more pronounced in the HDM-MSC-EPA mice. In addition, Est,L was reduced in HDM-MSC-EPA, but not in HDM-MSC mice.

### EPA Did Not Enhance MSC Engraftment in Lung Tissue

Three days after MSCs administration, very little Y-chromosome DNA was detected in lung tissue in the HDM-MSC and HDM-MSC-EPA mice with no differences between the groups (Figure S6 in Supplementary Material).

## Discussion

In the model of HDM-induced allergic asthma used herein, EPA-stimulated MSCs, compared to unstimulated MSCs, yielded greater therapeutic effects by further reducing bronchoconstriction, alveolar collapse, total cell count in BALF, bone marrow, and lymph nodes, and collagen fiber content in airways, while increasing BALF IL-10 levels and M2 macrophage counts in lungs. Furthermore, in the *in vitro* experiments, EPA-stimulated MSCs exhibited an increase in LB count and in the levels of IL-10, TGF-β1, RvD_1_, and PGE_2_ secreted compared to unstimulated MSCs.

Although ovalbumin is widely used to induce allergic asthma in animal models, it requires peripheral sensitization and the use of adjuvants to obtain a successful experimental protocol (34). HDM has been considered a more clinically relevant allergen, as it affects ~85% of asthmatic patients worldwide and the experimental syndrome exhibit inflammatory (eosinophilia and Th2 pro-inflammatory cytokine increase) and ultrastructural changes in the airway and lung parenchyma, which closely mimic the hallmark features of human disease (13, 35). In our study, MSCs were administered 24 h after the last HDM challenge, when changes in lung mechanics, inflammation, and remodeling were already established. This contrasts with some previous studies, in which cell-based therapy was administered prophylactically (9–11, 36–38). In addition, MSCs were obtained from bone marrow, since this source has been shown to yield greater therapeutic responses compared to MSCs from other sources (7, 13). MSCs were administered intratracheally because cells would be directly delivered to the injured environment by this route, which could potentiate their therapeutic properties (39).

Mesenchymal stromal cells can secrete potent combinations of trophic factors that modulate cellular responses in the injured environment to induce anti-inflammatory and pro-resolution responses (6, 8, 40). In the present study, administration of unstimulated MSCs modulated BALF levels of IL-13, thereby decreasing total and differential cell count in BALF. In agreement with our findings, MSCs have been shown to mitigate inflammation by reducing secretion of pro-inflammatory mediators in different models of allergic asthma and at different levels of severity (7, 9–13, 36–38).

Even though both systemic and intratracheal administration of MSCs results in significant anti-inflammatory effects in the lungs, these effects are limited in terms of ability to repair tissue damage and revert the remodeling process (7, 12, 13). Therefore, recent studies have attempted to potentiate MSCs actions *in vivo* through *in vitro* preconditioning with physical, biological, and/or chemical stimuli to enhance cell survival and regenerative properties, and boost the secretion of trophic factors (15). In this line, EPA may be an interesting MSC enhancer, as it modulates several aspects of inflammatory lipid mediator synthesis and activity (19, 41, 42). EPA may also serve as a substrate for the synthesis of 3-series prostaglandins and 5-series leukotrienes, which have lower agonistic activity at eicosanoid receptors than their arachidonic acid-derived counterparts (43). Sequential metabolism of EPA by 5-LO and 15-LO produces resolvins, which induce pro-resolution effects in asthma models when exogenously administered (44). Thus, exposure of MSCs to EPA could enhance the production of anti-inflammatory and pro-resolution mediators. In our *in vitro* experiments, EPA enhanced LB formation in MSCs, which suggests an increase in EPA mobilization to act as direct receptor ligands or for further metabolism into lipid mediators (31, 33). In addition, EPA enhanced IL-10, TGF-β, RvD_1_, and PGE_2_ secretion by MSCs, which may induce anti-inflammatory and pro-resolution responses in allergic asthma (19). As a proof-of-concept, we treated MSCs with i15-LO to block EPA metabolism (19, 41), which abrogated the increased secretion of RvD_1_ and PGE_2_ in EPA-stimulated MSCs.

Mobilization of omega-3 fatty acid-derived biosynthetic pathways enhances the generation of lipid-derived pro-resolution mediators, including RvD_1_, and accelerates the catabasis of Th2-mediated inflammation (45). In this line, EPA-stimulated MSCs reduced secretion of both IL-4 and IL-13 and further decreased total and differential cellularity in the BALF compared to unstimulated MSCs.

Increased secretion of PGE_2_ and IL-10 by EPA-stimulated MSCs may also help resolve the inflammatory process in HDM-induced allergic asthma. Previous studies have shown that PGE_2_-induced EP_4_ receptor activation mitigates airway inflammation (46) and IL-10 inhibits eosinophilia by suppressing Th2 cytokine production (47), which is in line with the anti-inflammatory effects of EPA-stimulated MSCs in HDM-challenge mice. Importantly, MSCs may alter macrophages metabolic status *vi*a a PGE_2_-dependent mechanism by promoting anti-inflammatory M2 rather than inflammatory M1 polarization (48); this, in turn, contributes to enhanced IL-10 secretion in the lungs (7, 11, 13, 48). In mouse models of Duchenne muscular dystrophy and myocardial infarction, EPA was described as an inhibitor of muscle damage through inhibition of M1 and promotion of M2 macrophage polarization when administered systemically (49, 50). Thus, EPA-stimulation of MSCs may provide a more targeted delivery of EPA and/or EPA-derived mediators to modulate macrophage polarization.

Several studies have indicated that the therapeutic effects of MSCs are independent of cell engraftment in the injured tissue (39, 51, 52). In particular, MSCs were found neither in mLNs nor in thymus and bone marrow of mice on the first and third day after MSCs administration *via* intratracheal route in the same model used in this study (13). Additionally, in this study, EPA stimulation did not enhance MSCs engraftment in lung tissue. It is possible that intratracheal administration of MSCs induced an anti-inflammatory response in the airways, which then had an indirect impact on inflammatory cell counts in other tissues.

IL-4 and IL-13 play important roles not only in the inflammatory process but also in lung remodeling by inducing fibroblast proliferation and increasing extracellular matrix deposition (53, 54). VEGF also contributes to tissue remodeling by increasing angiogenesis and vascular permeability, as well as smooth muscle cell hyperplasia (2, 55). Taken together, these factors led to alveolar collapse and impairment of lung function observed in HDM-challenge mice. EPA-stimulated MSCs administration reduced α-SMA expression and elastic and collagen fiber content in both lung parenchyma and airways in HDM-challenge mice, leading to a significant improvement in lung mechanics. Unstimulated MSCs similarly reduced elastic fiber deposition but were inefficient at reducing α-SMA expression and collagen fiber content, thus failing to improve lung function as much as EPA-stimulated MSCs. This fact may be attributed to the lower capacity of unstimulated MSCs of reducing secretion of pro-fibrotic mediators, including IL-4 and VEGF, in HDM-challenge mice. In addition, increased PGE_2_ secretion may inhibit α-SMA expression and myofibroblast differentiation (56). Previous studies have also shown that administration of unstimulated MSCs has only marginal ability to reverse the remodeling process, mainly in the airways (7, 12, 13), which is in line with our results.

Goblet cell hyperplasia and hypertrophy is also a major event in the remodeling process of asthma (2). HDM-challenged mice presented an increased bronchoconstriction index, which may be attributable both to mucus-producing cells and to smooth muscle cell hyperplasia and hypertrophy. IL-13 has been shown to drive epithelial cell differentiation in goblet cells and upregulate MUC5AC expression (57). In the current study, we observed increased IL-13 secretion and mucin-containing cell counts in HDM-challenged mice; however, the reduction in IL-13 secretion after administration of unstimulated MSCs was not accompanied by a reduction in mucus hypersecretion, which suggests the involvement of other mediators or pathways to resolve this morphological abnormality. In this context, increased secretion of RvD_1_ in EPA-stimulated MSCs may be implicated in the reduction of mucus-filled cell counts observed in HDM-challenge mice. This is supported by the finding that mucus hypersecretion decreased markedly after RvD_1_ was exogenously administered in a model of ovalbumin-induced allergic asthma (58).

This study has some limitations that should be addressed. First, total cell counts and levels of pro- and anti-inflammatory mediators were not evaluated in blood, as a mouse weighing 25 g has a total blood volume of approximately 1.2 mL, which would make such a wide range of analyses impossible. Nevertheless, the systemic effects of MSCs (intratracheally administered) were evaluated by quantifying total cell count in bone marrow, lymph nodes, and thymus. Second, the biodistribution of unstimulated and EPA-stimulated MSCs was not assessed, even though EPA did not alter MSCs homing.

## Conclusion

Eicosapentaenoic acid stimulation enhances the effects of MSC therapy in experimental allergic asthma, leading to increased secretion of pro-resolution and anti-inflammatory mediators, modulation of macrophages toward an anti-inflammatory phenotype, and reductions in elastic and collagen fiber content, α-SMA expression, and mucin-containing cell counts. Taken together, these modifications may explain the greater improvement in lung mechanics observed after administration of EPA-stimulated MSCs in HDM-challenged mice. This may be a promising novel strategy for MSCs potentiation.

## Ethics Statement

This study was approved by the Animal Care and Use Committee (CEUA: 018-14) of the Health Sciences Center, Federal University of Rio de Janeiro, Rio de Janeiro, Brazil (chair: Prof. M. Frajblat). All animals received humane care in compliance with the “Principles of Laboratory Animal Care” formulated by the National Society for Medical Research and the U.S. National Academy of Sciences Guide for the Care and Use of Laboratory Animals.

## Author Contributions

SA and ML-P: contributed to overall study design, experimental activities, data analysis and interpretation, and writing of the manuscript. AS: contributed to experimental activities, data analysis and interpretation, and writing of the manuscript. DX, TO, JK, LC, NA, VM, LS, and CG-d-A: contributed to experimental activities and data analysis and interpretation. HC-F-N, PO, DW, and MM: contributed to overall study design, data analysis and interpretation, and writing of the manuscript. BD and PR: contributed to overall study design, experimental activities, data analysis and interpretation, writing of the manuscript, and provided financial support. All authors approved the final version of the manuscript.

## Conflict of Interest Statement

The authors declare that the research was conducted in the absence of any commercial or financial relationships that could be construed as a potential conflict of interest.
