# Supplementary material for: Eicosapentaenoic Acid Enhances the Effects of Mesenchymal Stromal Cell Therapy in Experimental Allergic Asthma
Source: Front Immunol. 2018 May 24;9:1147. doi: 10.3389/fimmu.2018.01147 (PMC5976792; doi:10.3389/fimmu.2018.01147)
Supplement: Supplementary file 2 [file Image_2.PDF]

Supplementary Material

Eicosapentaenoic acid enhances the effects of mesenchymal stromal cell therapy in experimental allergic asthma

Soraia Carvalho Abreu, Miquéias Lopes-Pacheco, Adriana Lopes da Silva, Debora Gonçalves Xisto, Tainá Batista de Oliveira, Jamil Zola Kitoko, Lígia Lins de Castro, Natália Recardo Amorim, Vanessa Martins, Luisa H. A. Silva, Cassiano Felippe Gonçalves-de-Albuquerque, Hugo Caire de Castro Faria-Neto, Priscilla Christina Olsen, Daniel Jay Weiss, Marcelo Marcos Morales, Bruno Lourenço Diaz, Patricia Rieken Macêdo Rocco

Correspondence: Patricia R M Rocco, M.D., PhD: prmrocco@gmail.com

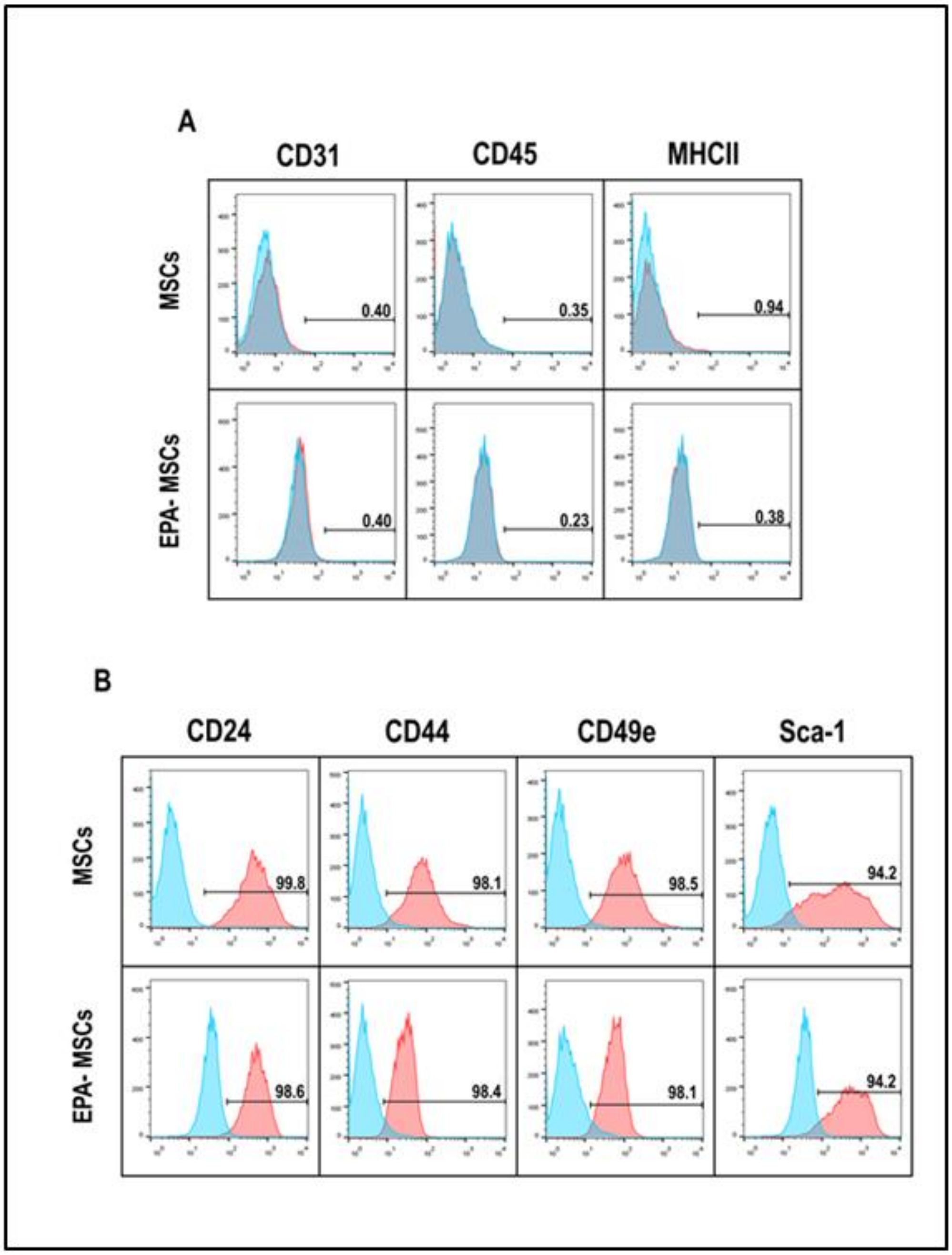

Supplementary Figure 2. Flow cytometry analysis. Unstimulated (MSCs) and EPA-stimulated MSCs (EPA-MSCs). (A) CD31−, CD45−, and MHCII− cells. (B) CD24+, CD44+, CD49e+, and Sca1+ cells.
