# Supplementary material for: Eicosapentaenoic Acid Enhances the Effects of Mesenchymal Stromal Cell Therapy in Experimental Allergic Asthma
Source: Front Immunol. 2018 May 24;9:1147. doi: 10.3389/fimmu.2018.01147 (PMC5976792; doi:10.3389/fimmu.2018.01147)
Supplement: Supplementary file 4 [file Image_4.PDF]

Supplementary Material

Eicosapentaenoic acid enhances the effects of mesenchymal stromal cell therapy in experimental allergic asthma

Soraia Carvalho Abreu, Miquéias Lopes-Pacheco, Adriana Lopes da Silva, Debora Gonçalves Xisto, Tainá Batista de Oliveira, Jamil Zola Kitoko, Lígia Lins de Castro, Natália Recardo Amorim, Vanessa Martins, Luisa H. A. Silva, Cassiano Felippe Gonçalves-de-Albuquerque, Hugo Caire de Castro Faria-Neto, Priscilla Christina Olsen, Daniel Jay Weiss, Marcelo Marcos Morales, Bruno Lourenço Diaz, Patricia Rieken Macêdo Rocco

Correspondence: Patricia R M Rocco, M.D., PhD: prmrocco@gmail.com

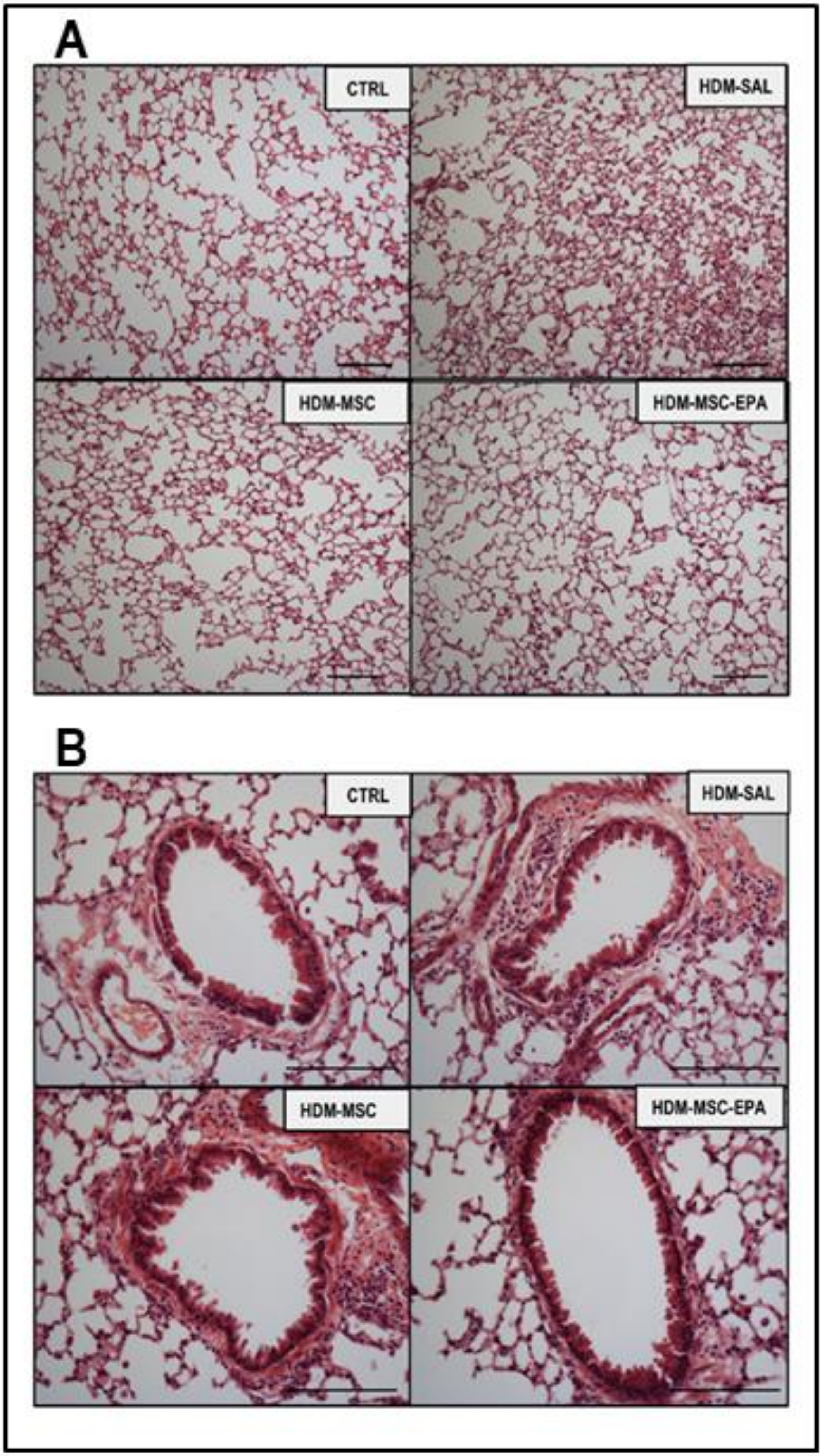
A
CTRL
HDM-SAL
HDM-MSC
HDM-MSC-EPA
B
CTRL
HDM-SAL
HDM-MSC
HDM-MSC-EPA

Supplementary Figure 4. Lung histology. Representative photomicrographs of lung parenchyma **(A)** and airways **(B)** stained with hematoxylin-eosin. Collapsed areas in the lung parenchyma and a reduction in central airway diameter, as well as increased cell infiltration, are visible in the HDM group. CTRL: saline-challenge mice; HDM: HDM-challenge mice; SAL: mice treated with saline; MSC: mice treated with unstimulated MSCs; HDM-EPA: mice treated with EPA-stimulated MSCs. Original magnification: ×200 **(A)** and ×400 **(B)**. Bar = 100 μm.
