# Supplementary material for: Eicosapentaenoic Acid Enhances the Effects of Mesenchymal Stromal Cell Therapy in Experimental Allergic Asthma
Source: Front Immunol. 2018 May 24;9:1147. doi: 10.3389/fimmu.2018.01147 (PMC5976792; doi:10.3389/fimmu.2018.01147)
Supplement: Supplementary file 5 [file Image_5.PDF]

Supplementary Material

Eicosapentaenoic acid enhances the effects of mesenchymal stromal cell therapy in experimental allergic asthma

Soraia Carvalho Abreu, Miquéias Lopes-Pacheco, Adriana Lopes da Silva, Debora Gonçalves Xisto, Tainá Batista de Oliveira, Jamil Zola Kitoko, Lígia Lins de Castro, Natália Recardo Amorim, Vanessa Martins, Luisa H. A. Silva, Cassiano Felippe Gonçalves-de-Albuquerque, Hugo Caire de Castro Faria-Neto, Priscilla Christina Olsen, Daniel Jay Weiss, Marcelo Marcos Morales, Bruno Lourenço Diaz, Patricia Rieken Macêdo Rocco

Correspondence: Patricia R M Rocco, M.D., PhD: prmrocco@gmail.com

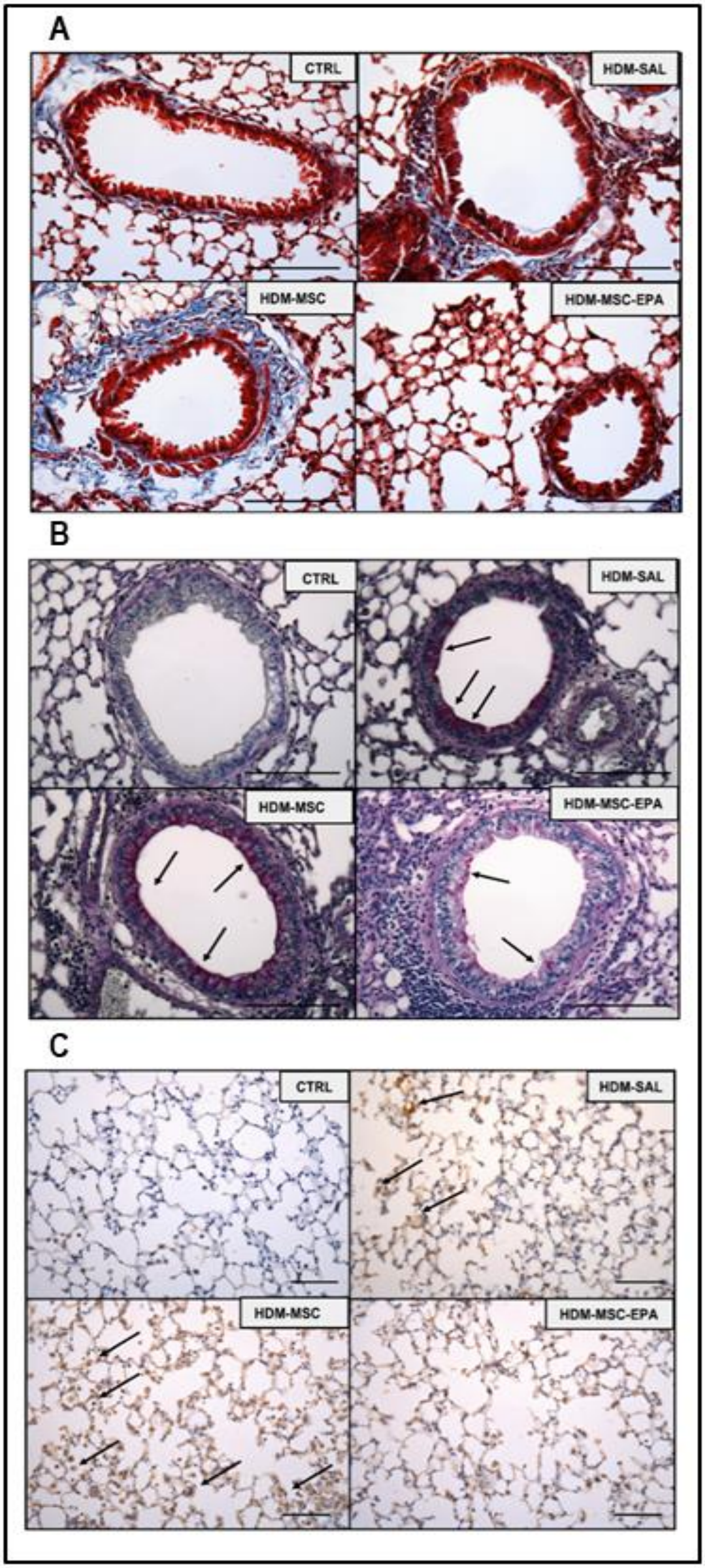
A
CTRL
HDM-SAL
HDM-MSC
HDM-MSC-EPA
B
CTRL
HDM-SAL
HDM-MSC
HDM-MSC-EPA
C
CTRL
HDM-SAL
HDM-MSC
HDM-MSC-EPA

Supplementary Figure 5. Lung morphological changes. Representative photomicrographs of **(A)** airways stained with Masson's Trichome, **(B)** airways stained with periodic acid-Schiff, and **(C)** immunohistochemical staining for smooth-muscle-specific α-actin in lung tissue. In **A**, marked deposition of collagen fibers is visible in blue around the airways in the HDM and HDM-MSC groups. In **B**, there is mucus hypersecretion in the airways (stained purple, arrows) in HDM and HDM-MSC groups. In **C**, note positive staining for α-actin (brown dots, arrows) in HDM and HDM-MSC. CTRL: saline-challenge mice; HDM: HDM-challenge mice; SAL: mice treated with saline; MSC: mice treated with unstimulated MSCs; HDM-EPA: mice treated with EPA-stimulated MSCs. Original magnification: ×400 (A, B) and ×200 (C). *Bar* = 100 μm. Analysis was performed in 8 specimens from each group.
