# Supplementary material for: Eicosapentaenoic Acid Enhances the Effects of Mesenchymal Stromal Cell Therapy in Experimental Allergic Asthma
Source: Front Immunol. 2018 May 24;9:1147. doi: 10.3389/fimmu.2018.01147 (PMC5976792; doi:10.3389/fimmu.2018.01147)
Supplement: Supplementary file 6 [file Image_6.PDF]

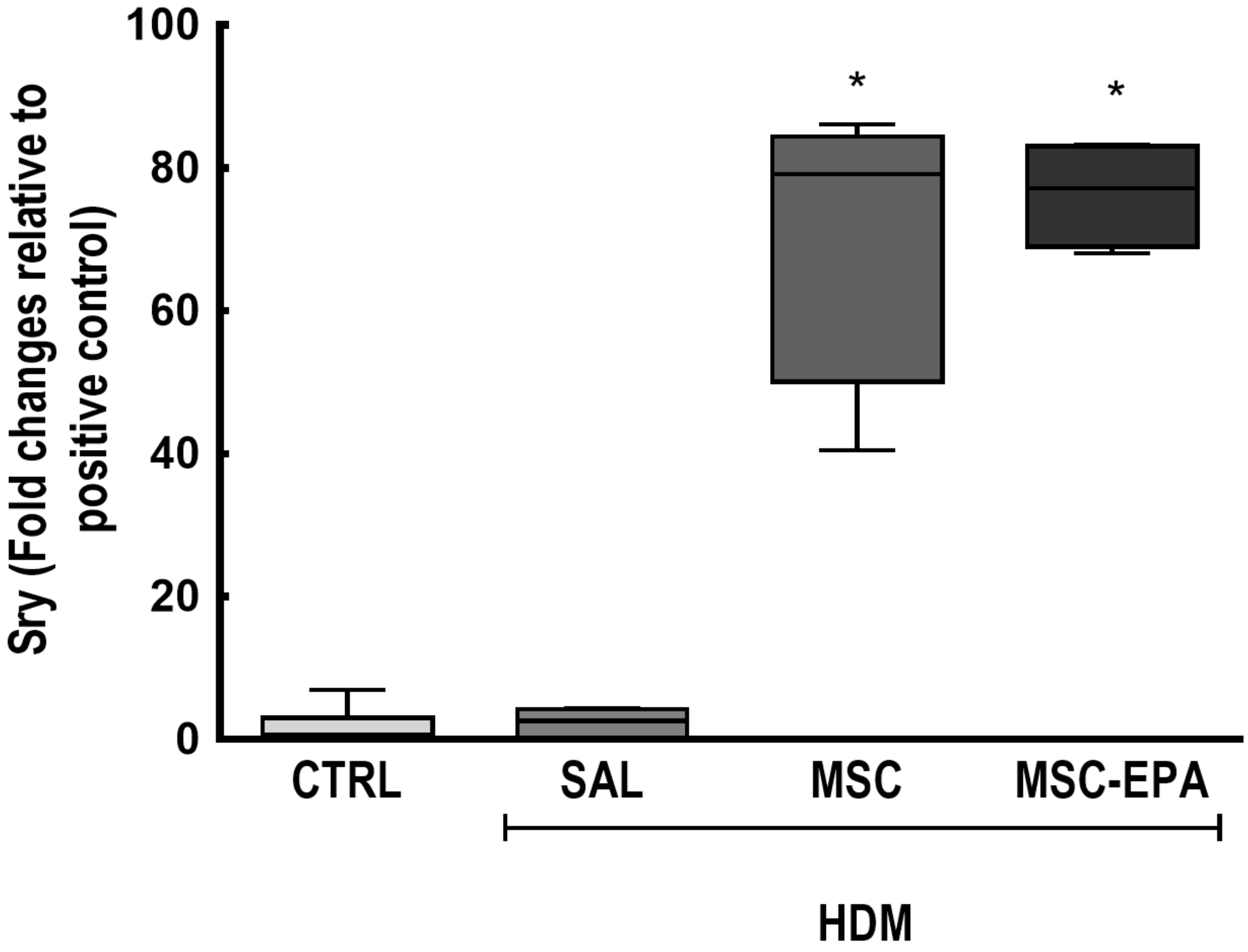
Sry (Fold changes relative to positive control)
100
80
60
40
20
0
*
*
CTRL
SAL
MSC
MSC-EPA
HDM

Supplemental Figure 6. Quantification of Y-chromosome DNA in lung tissue 3 days after intratracheal administration of unstimulated (MSC) and EPA-stimulated mesenchymal stromal cells (MSC-EPA). CTRL, saline-challenged mice; HMD, HMD-challenged mice; SAL, HDM mice treated with saline; MSC, HDM mice treated with unstimulated MSCs; MSC-EPA, HDM mice treated with EPA-stimulated MSCs. Boxes show the interquartile (25–75%) range, whiskers denote the range (minimum–maximum), and horizontal lines represent the median of 5 animals/group. * Significantly different from CTRL ($p<0.05$).
